# Impact of upfront randomization for postoperative treatment on quality of surgery in the CRITICS gastric cancer trial

**DOI:** 10.1007/s10120-018-0875-1

**Published:** 2018-09-20

**Authors:** Y. H. M. Claassen, H. H. Hartgrink, W. O. de Steur, J. L. Dikken, J. W. van Sandick, N. C. T. van Grieken, A. Cats, A. K. Trip, E. P. M. Jansen, W. M. Meershoek-Klein Kranenbarg, J. P. B. M. Braak, H. Putter, M. I. van Berge Henegouwen, M. Verheij, C. J. H. van de Velde

**Affiliations:** 1 Department of Surgical Oncology, Leiden University Medical Center, Leiden, The Netherlands; 2 Department of Surgical Oncology, The Netherlands Cancer Institute, Antoni van Leeuwenhoek Hospital, Amsterdam, The Netherlands; 3 Department of Pathology, VU University Medical Center, Amsterdam, The Netherlands; 4 Department of Gastrointestinal Oncology, The Netherlands Cancer Institute, Antoni van Leeuwenhoek Hospital, Amsterdam, The Netherlands; 5 Department of Radiation Oncology, The Netherlands Cancer Institute, Antoni van Leeuwenhoek Hospital, Amsterdam, The Netherlands; 6 Department of Medical Statistics, Leiden University Medical Center, Leiden, The Netherlands; 7 Department of Surgery, Academic Medical Center, Amsterdam, The Netherlands

**Keywords:** Gastric cancer surgery, Surgical quality, Upfront randomization

## Abstract

**Background:**

Preoperative randomization for postoperative treatment might affect quality of surgery. In the CRITICS trial (ChemoRadiotherapy after Induction chemotherapy In Cancer of the Stomach), patients were randomized before treatment to receive chemotherapy prior to a D1 + gastrectomy (removal of lymph node station (LNS) 1–9 + 11), followed by either chemotherapy (CT) or chemoradiotherapy (CRT). In this analysis, the influence of upfront randomization on the quality of surgery was evaluated.

**Methods:**

Quality of surgery was analyzed in both study arms using surgicopathological compliance (removal of ≥ 15 lymph nodes), surgical compliance (removal of the indicated LNS), and surgical contamination (removal of LNS that should be left in situ). Furthermore, the ‘Maruyama Index of Unresected disease’ (MI) was evaluated in both study arms, and validated with overall survival.

**Results:**

Between 2007 and 2015, 788 patients with gastric cancer were included in the CRITICS study of which 636 patients were operated with curative intent. No difference was observed between the CT and CRT group regarding surgicopathological compliance (74.8% vs 70.9%, *P* = 0.324), surgical compliance (43.2% vs 39.2%, *P* = 0.381), and surgical contamination (59.4% vs 59.9%, *P* = 0.567). Median MI was 1 in both groups (range CT 0–88 and CRT 0–136, *P* = 0.700). A MI below 5 was associated with better overall survival (CT: *P* = 0.009 and CRT: *P* = 0.013).

**Conclusion:**

Surgical quality parameters were similar in both study arms in the CRITICS gastric cancer trial, indicating that upfront randomization for postoperative treatment had no impact on the quality of surgery. A Maruyama Index below five was associated with better overall survival.

## Introduction

Timing of randomization in multimodality trials is often a point of debate. This is illustrated by the criticism on the timing of randomization in the Intergroup 0116 trial where randomization for adjuvant chemoradiotherapy versus no adjuvant treatment was done after surgery [1]. Opponents found that this moment of randomization may have led to selection bias, as pathology results were known at the time of selecting patients for the study. Preoperative randomization avoids this patients’ selection for study participation after surgery.

**Fig. 1:**
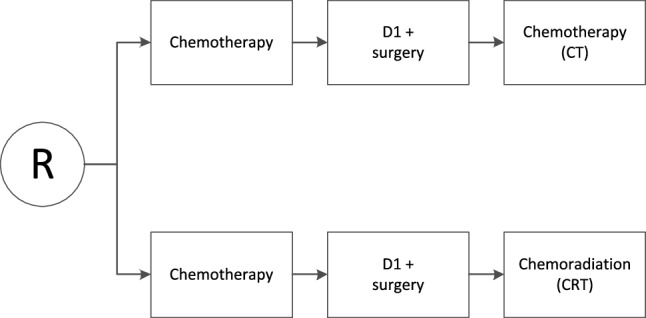
Design of the CRITICS trial. *R* randomization, *Chemotherapy* epirubicin, cisplatin/oxaliplatin, and capecitabine (ECC/EOC), *D1 + surgery* surgery including a D1 + lymphadenectomy, *Chemoradiotherapy* 45 Gy/25 fractions + capecitabine + cisplatin

The US Intergroup 0116 trial and the British MAGIC trial changed the current clinical practice for resectable gastric cancer in the Western world, by showing a survival benefit with adjuvant chemoradiotherapy and perioperative chemotherapy, respectively [1, 2]. As the results of the Intergroup 0116 trial and the MAGIC trial were not directly comparable due to differences in study design and eligibility criteria, the CRITICS trial (ChemoRadiotherapy after Induction chemotherapy In Cancer of the Stomach) was initiated. In this multicenter trial, patients with resectable gastric cancer were treated with three cycles of preoperative chemotherapy and surgery with an adequate lymph node dissection, followed by either three cycles of chemotherapy (CT) or concurrent chemoradiotherapy (CRT). Randomization was done before the start of preoperative chemotherapy (Fig. 1) [3]. The moment of randomization has been criticized. It has been suggested that the quality of surgery in the CRITICS study might be influenced by the knowledge of the treatment that would follow, as surgeons were not blinded for the adjuvant therapy. To dispel this assumption, the possible influence of upfront randomization for the postoperative treatment on the quality of surgery in the CRITICS trial was investigated in the current analyses.

Surgical quality was assessed in both study arms using surgicopathological compliance (removal of at least 15 lymph nodes), surgical compliance (removal of the indicated lymph node stations), and surgical contamination (removal of lymph node stations that should be left in situ). Furthermore, surgical quality was analyzed by calculating the ‘Maruyama Index of Unresected disease’ (MI), the strongest quality indicator for determining the adequacy of lymphadenectomy in gastric cancer surgery. Additionally, the MI was validated with overall survival, as in both the Dutch Gastric Cancer Trial (DGCT) and the Intergroup 0116 trial, the MI proved to be strongly associated with survival, with a cut-off value below five for a favorable outcome [4–6]. By analyzing these surgical quality parameters in both study arms the aim of the current study was to evaluate the possible influence of upfront randomization for postoperative treatment on the quality of surgery in the CRITICS gastric cancer trial.

## Methods

### CRITICS protocol

The study protocol of the CRITICS trial has been published previously [3]. Patients with a histologically proven stage Ib-IVa (AJCC 6th edition) gastric adenocarcinoma were included [7]. The bulk of the tumor had to be located in the stomach, though extension into the gastro-esophageal junction (GEJ) was allowed. Patients with ASA classification 1 or 2 were included. The most important exclusion criteria were inoperability, distant metastases, and T1N0 disease (determined with endoscopic ultrasound).

Prior to surgery, all patients were assigned to receive three cycles of epirubicin, cisplatin/oxaliplatin, and capecitabine (ECC/EOC) at 3-weekly intervals. Surgery was scheduled 3–6 weeks after the last chemotherapy cycle. The principle of surgery was a wide resection of the tumor bearing part of the stomach with en bloc removal of lymph nodes at stations 1–9 and 11 (D1 + lymph node dissection) and with a minimum of 15 lymph nodes. A D1 + was chosen with best insight while the discussion regarding the extent of lymphadenectomy for gastric cancer in the Western world was still ongoing at the moment of designing the trial. A D1 lymph node dissection was defined as removal of stations 3–6 during subtotal gastrectomy and stations 1–6 during total gastrectomy. A D2 lymph node dissection was defined as removal of stations 1,3, 4sb, 4d, 5, 6, 7, 8a, 9, 11p, and 12a during subtotal gastrectomy and stations 1–7, 8a, 9, 10, 11p, 11d, and 12a during total gastrectomy [8]. Adjacent organs were only removed if there was suspicion of tumor involvement. If possible, a macroscopic margin of 5 cm was obtained, both to the proximal end and to the distal end. For tumors in the upper part of the stomach, a total gastrectomy was performed. For tumors in the middle or distal part of the stomach, a subtotal resection of the stomach was performed, leaving lymph node stations 2 and 4 s in situ. A transhiatal esophagus-cardia resection with gastric tube reconstruction was performed for gastro-esophageal junction (GEJ) tumors extending into the esophagus, leaving lymph node stations 4d and 6 in situ. Both open and minimally invasive procedures were allowed.

After surgery, the study protocol dictated either another three courses of ECC/EOC (CT) or chemoradiotherapy (CRT; 45 Gy in 25 fractions combined with daily capecitabine and weekly cisplatin). Randomization of the adjuvant therapy occurred prior to the start of treatment (Fig. 1).

### Surgical quality assurance in the CRITICS trial

Before participation in the CRITICS trial, a presentation was given to instruct surgeons which lymph node stations had to be removed according to the study protocol. Participating surgeons also received a DVD and a book with instructions as well. Continuous quality assurance was carried out since 2011 and included regular feedback to the participating surgeon and pathologist on their average lymph node count in the trial, together with the average lymph node count in the study at that moment. In addition, if the study coordinator received a report with a lymph node count below 15, feedback was provided within 3 months after surgery to the respective surgeon and pathologist and if possible, the surgical specimen was examined for remaining lymph nodes.

### Eligibility current study

For the current analyses, patients were selected from the CRITICS database if the gastric cancer operation was performed with curative intent, based on the surgical report.

Patients were excluded from the surgicopathological analyses if the total number of sampled lymph nodes was not documented by the pathologist. Patients were excluded from the analyses of surgical compliance, surgical contamination, and MI, if the exact location of the directed lymph node stations was not extractable from the surgical report.

This study was reported according the CONSORT 2010 statement [9].

### Central data review

Data on the dissected lymph node stations (1–16) and type of lymph node dissection (D1 + or more) were extracted from the surgical reports, supplementary to the data recorded in the CRF. These data were validated and optimized by two experienced gastric surgeons. In case the number of removed lymph node stations was not explicitly stated in the surgical report, an assumption was made based on the mentioned anatomical structures in the surgical report, if possible. For example, when a given surgical report described the removal of lymph nodes along the common hepatic artery, it was revised as the removal of lymph node station 8. If assumptions were not possible, it was scored as unknown. In case all stations were unknown, patients were excluded from the analyses. In case a single lymph node station was unknown, the station was considered as not removed.

### Surgicopathological compliance

Surgicopathological compliance was defined as the removal of a minimum of 15 lymph nodes and surgicopathological non-compliance as the removal of less than 15 lymph nodes. The latter group was divided into minor surgicopathological non-compliance, defined as the removal of a minimum of 10 lymph nodes, and major surgicopathological non-compliance, defined as the removal of less than 10 lymph nodes.

### Surgical compliance and surgical contamination

Surgical compliance was defined as the removal of stations 1–9 and 11, except for subtotal gastric resections where lymph node stations 2 and 4 s were left in situ, and esophagus-cardia resections where lymph node stations 4d and 6 were left in situ. The definition of surgical non-compliance was not harvesting all indicated lymph node stations. The surgical non-compliance group was divided into minor non-compliance (1 or 2 of the intended lymph node stations not removed) and major non-compliance (≥ 3 of the intended lymph node stations not removed).

Surgical contamination was defined as removal of one or more lymph node stations outside the intended extent of resection. Surgical contamination was subdivided into minor contamination (1 or 2 lymph node stations that should be left in situ removed) and major contamination (≥ 3 lymph node stations that should be left in situ removed). Surgical compliance and surgical contamination were based on the data validated by two experienced gastric surgeons.

### Maruyama index

The MI is based on eight parameters: sex, age, type of cancer (early or advanced), depth of invasion, maximal diameter, location (upper third, middle third, lower third), position (lesser curvature, greater curvature, anterior, posterior, circular), and histological type. In the current study, the MI was determined using the Maruyama Computer Program. To quantify the likelihood of unresected nodal disease, the MI is defined as the sum of Maruyama Computer Program predictions for the regional lymph node stations 1–12, which were not removed by the surgeon. When a given patient underwent a total gastrectomy with removal of lymph node stations 1–7 and 9, the MI was calculated by adding up the likelihood of unresected nodal disease at stations 8, 10, 11, and 12.

### Statistics

The chi-squared test was used to compare categorical data between the CT and CRT group and the unpaired *t* test was used for numerical data. Overall survival since surgery for both study arms was estimated by the Kaplan–Meier method and survival distribution of MI (< 5 and ≥ 5) was assessed by the log-rank test. The effect of MI (< 5 and ≥ 5) on survival in both groups was determined by an interaction test. A *P* lower than 0.05 was considered as statistically significant. SPSS program 21.0 was used for statistical analyses.

## Results

**Fig. 2:**
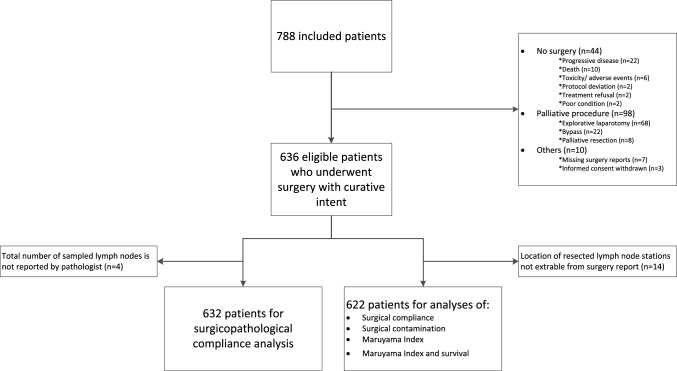
Study flow chart

From January 2007 to April 2015, 788 patients were included at 56 centers in the Netherlands, Sweden, and Denmark. For current analyses, 636 patients were eligible; 632 patients for the analyses on surgicopathological compliance, 622 patients for the analyses on surgical compliance, surgical contamination, MI, and MI and survival (Fig. 2).

Patient characteristics are shown in Table 1. The localization of the primary tumor (proximal, middle, distal stomach) was equally distributed in the CT group and in the CRT group. In both groups, the majority of patients underwent a total gastrectomy, followed by a subtotal gastrectomy, and an esophagus-cardia resection. In the CT group, 22 patients underwent a splenectomy (7.1%) compared to 16 patients (4.9%) in the CRT group. The rate of distal pancreatectomies was low in both groups, 6 patients (1.9%) in the CT group and 10 patients (3.1%) in the CRT group, respectively.

**Table 1:** Patient characteristics

	CT group (*n* = 310)	CRT group (*n* = 326)	*P*
Median age (years)	61.5(28–81)	63.0 (30–82)	0.240
Sex			
Male	214 (69.0)	215 (66.0)	0.359
Female	96 (31.0)	111 (34.0)	
Lauren classification			
Diffuse	101 (32.6)	105 (32.2)	0.712
Intestinal	88 (28.4)	87 (26.7)	
Mixed	13 (4.2)	21 (6.4)	
Unknown	108 (34.8)	112 (34.7)	
Tumor localization			
Proximal stomach	116 (37.8)	120 (36.8)	0.655
Middle stomach	95 (30.7)	88 (27.0)	
Distal stomach	99 (31.6)	118 (36.2)	
Type of resection			
Total gastrectomy	159 (51.3)	159 (48.8)	0.688
Subtotal gastrectomy	119 (38.4)	136 (41.7)	
Esophagus-cardia resection	32 (10.3)	31 (9.5)	
Tumor stage			
pT0/pTis/pT1	62 (20.0)	71 (21.8)	0.882
pT2	108 (34.8)	114 (35.0)	
pT3	110 (35.5)	107 (32.8)	
pT4	30 (9.7)	34 (10.4)	
Node stage			
pN0	150 (48.4)	161 (49.4)	0.846
pN1	109 (35.1)	105 (32.2)	
pN2	35 (11.3)	42 (12.9)	
pN3	16 (5.2)	18 (5.5)	
UICC stage			
Stage 0	21 (6.8)	22 (6.7)	0.373
Stage 1	100 (32.3)	101 (31.0)	
Stage 2	65 (21.0)	84 (25.8)	
Stage 3	87 (28.1)	73 (22.4)	
Stage 4	37 (11.9)	46 (14.1)	
Splenectomy			
Yes	22 (7.1)	16 (4.9)	0.244
No	288 (92.9)	310 (95.1)	
Distal pancreatectomy			
Yes	6 (1.9)	10 (3.1)	0.489
No	304 (98.1)	316 (96.9)	
Approach			
Open	256 (82.6)	274 (84.0)	0.837
Minimally invasive	46 (14.8)	43 (13.2)	
Conversion	6 (1.9)	6 (1.8)	
Unknown	2 (0.7)	3 (1.0)	
Surgical complication			
Yes	70 (22.6)	72 (22.1)	0.880
No	240 (77.4)	254 (77.9)	
Median *n*# LN dissected	21 (0–72)	19 (0–71)	0.037
Radicality			
R0	248 (80.0)	267 (81.9)	0.828
R1	34 (11.0)	32 (9.8)	
Unknown	28 (9.0)	27 (8.3)	

Age and median *n*# of LN dissected is presented as median (range), other data are presented as *n* (%)

*CT group* chemotherapy, *CRT group* chemoradiotherapy, *median n# LN dissected* median number of lymph nodes dissected

**Fig. 3:**
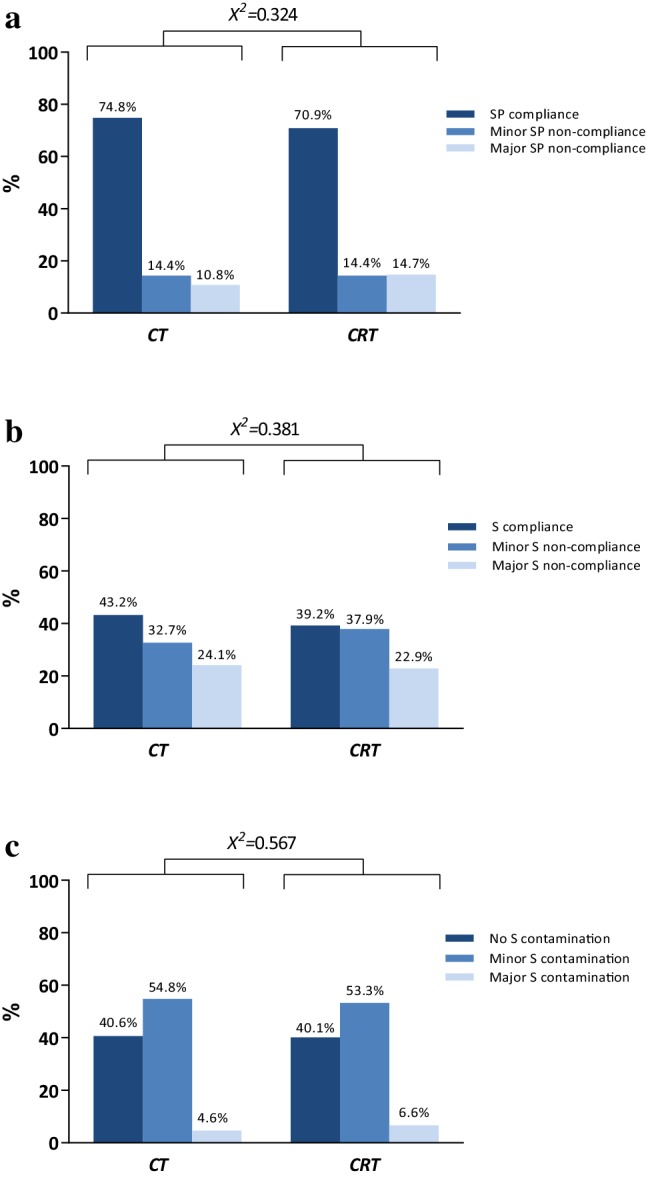
Comparison of the CT and the CRT group with regards to **a** surgicopathological (SP) compliance (≥ 15 lymph nodes), **b** surgical (S) compliance, and **c** surgical (S) contamination. *CT* chemotherapy, *CRT* chemoradiotherapy

Surgicopathological compliance occurred in 230 patients (74.8%) in the CT group and 232 patients (70.9%) in the CRT group (*P* = 0.324, Fig. 3a). Surgicopathological compliance improved over time both in the CT group (from 60.0 to 100%) and in the CRT group (from 50.0 to 80.0%). No significant difference was observed between the CT group and the CRT group with respect to at least a D1 + lymphadenectomy performed (88.8% vs 86.2%, *P* = 0.333). Complete surgical compliance occurred in 131 patients (43.2%) in the CT group and in 125 patients (39.2%) in the CRT group (*P* = 0.381, Fig. 3b). Similarly, surgical contamination was not different between the two study arms (Fig. 3c).

**Fig. 4:**
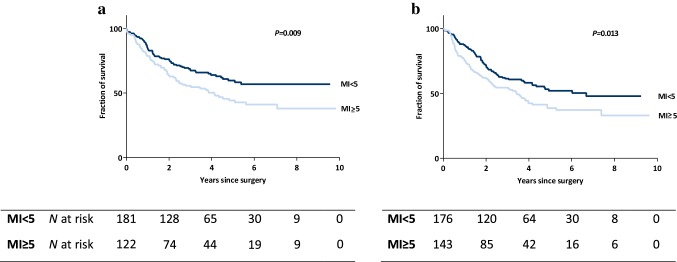
The Maruyama Index (MI) showing a statistically significant difference in overall survival between MI < 5 versus MI ≥ 5, both in the CT group (**a**) and in the CRT group (**b**). *CT* chemotherapy, *CRT* chemoradiotherapy, *N at risk* number of patients at risk

Median MI was 1 in both the CT group (range 0–88) and the CRT group (range 0–136, *P* = 0.700). A MI < 5 was associated with an improved overall survival in both groups (Fig. 4). The effect of MI < 5 on survival did not differ between the two groups (HR: 1.06; 95% CI: 0.67–1.69; *P* = 0.793).

## Discussion

In the CRITICS trial, gastric cancer patients were randomized before start of the treatment between adjuvant chemotherapy (CT) and adjuvant chemoradiotherapy (CRT) after preoperative chemotherapy and surgery. In the current study, the potential effect of upfront randomization for postoperative treatment on the quality of surgery was evaluated. No significant differences were observed between the CT and the CRT group with regard to a number of surgical quality parameters. A Maruyama Index, one of the most potent quality parameters in gastric cancer surgery, below 5 was associated with an improved overall survival in both groups.

The CRITICS trial was designed based on two randomized trials, the Intergroup 0116 trial and the MAGIC trial that changed current clinical practice in the Western world for locally advanced resectable gastric cancer by showing an improved survival with postoperative chemoradiotherapy and perioperative chemotherapy, respectively [1, 2]. In the Intergroup 0116 trial, patients were randomized 20–40 days after surgery, for postoperative chemoradiotherapy versus no adjuvant treatment. The study has been criticized for the fact that only 10% of the patients underwent the intended D2 lymph node dissection [1]. In the CRITICS trial in 73% of the patients, at least 15 lymph nodes were removed and around 41% of the patients underwent the intended D1 + dissection (surgical compliance) [10]. Although the latter finding is an improvement compared to the number of the Intergroup 0116 trial, surgical compliance in the CRITICS trial might have been expected to be higher due to the strict quality assurance program within the trial. However, when interpreting the surgical compliance rate in the CRITICS trial some aspects should be taken into account. First, surgical compliance is probably an underestimation, as ‘unknown lymph node station’ was considered ‘not removed’. Furthermore, in contrast to the Eastern world, lymph nodes of different lymph node stations in the Western world are not separately removed by the surgeon. As a consequence, the removal of specific lymph node stations is less recorded in surgery reports and all lymph nodes together are offered to the pathologist instead of lymph nodes from each specific lymph node station. The number of lymph nodes is therefore probably of more value than the surgical compliance rate.

Postoperative randomization such as in the Intergroup 0116 trial harbors the risk of selection bias, as only a proportion of patients will be able to start postoperative treatment. These patients may reflect a selection of younger, physically more fit patients with a good performance status, leading to a possible overestimation of the survival benefit. The extent of this selection will be considerable because it is known that after gastric cancer surgery a significant proportion of patients will never start, due to disease progression, postoperative complications, poor condition, refusal of patients, or even death. In the CRITICS trial, 61% of the patients in the CT group and 63% in the CRT group started postoperative treatment and 47% (CT group) and 54% (CRT group) was able to complete adjuvant therapy, respectively [11]. This is comparable to other gastric cancer trials as the Intergroup 0116 trial and the French FNCLCC and FFCD trial where 63% and 50% of the patients completed treatment according the study protocol, respectively [1, 12]. In the MAGIC trial, 66% of the patients commenced with postoperative chemotherapy and 43% of the patients managed to complete adjuvant treatment [2]. In this trial, patients were randomly assigned to either perioperative chemotherapy and surgical resection or to surgical resection alone, 6 weeks prior to surgery. With this design, insight is gained in the whole chain of multimodal treatments, thus more accurate information can be given to the patients’ options. This applies for randomized clinical trials with multimodal treatment routes; in general, however, in gastric cancer trials, this is even more important because the proportion of patients who do not complete the whole chain is substantial.

In the CRITICS trial, as in the MAGIC trial, patients were randomized for postoperative treatment before the start of treatment; either three additional courses of chemotherapy or chemoradiotherapy. It was decided to randomize prior to preoperative treatment to prevent selection of patients after surgery, which might bias the inclusion. Opponents have considered the preoperative randomization as a possible limitation of the CRITICS trial for the reason that this could influence the quality of surgery. These assumptions suggest that the surgical performance was influenced by the knowledge of the result of the randomization, as participating surgeons were not blinded for the adjuvant treatment. For instance, a surgeon might decide to perform a more extended lymphadenectomy in case a patient was randomized for ‘only’ chemotherapy instead of chemoradiotherapy.

Results of the current study showed no significant differences between the CRT and the CT group with regard to surgicopathological compliance, number of adequate lymphadenectomies performed, surgical compliance, and surgical contamination. Both groups had a median MI of 1. Altogether, there are no indications that upfront randomization for postoperative treatment in the CRITICS trial was associated with differences in the quality of surgery. Thereby, the primary outcomes of the CRITICS trial, overall survival and progression-free survival, can be compared more reliably between both arms, whereby more trustworthy conclusions can be drawn about the possible added effect of adjuvant therapy in patients with locally advanced resectable gastric cancer.

In the MAGIC trial, detailed information about the quality of surgery was lacking, and in the Intergroup 0116 trial, the proportion of adequate gastric cancer resections was low. The strength of the current study was the very detailed information on the quality of surgery, and it shows the success of the surgical quality assurance within the CRITICS trial.

The design of the CRITICS trial, including the upfront randomization, has its limitations. Inherent to this design, the number of randomized patients who completed the full multimodal treatment was around 50%, in both arms, leading to a possible underestimation of the treatment effect. On the other hand, this design provides insight in the entire chain of multimodal treatments for gastric cancer patients and reflects daily practice in treating Western gastric cancer patients.

In conclusion, our analyses indicate that upfront randomization for postoperative treatment did not influence the quality of surgery in the CRITICS trial. A Maruyama Index below five was associated with a better survival.
